# Cutaneous Tuberculosis

**Published:** 1919-03-29

**Authors:** 


### CUTANEOUS TUBERCULOSIS.

### A Review of Modern Treatment.

SKIN tubercle has always been a rather refractory thing to treat. Pyogenic scrofulosis and scrofulodermia take a good time in healing, and break down again most readily. For early lupus surgery did much, does much now, in fact, for many dermatologists aver that for a few nodules nothing beats " a good scrape." But in spite of this some cases became progressive, and constituted a sad reproach to therapeutics. Then when the old tuberculin was introduced, one of its rare successes was the occasional curing of lupus. As we can see now, with the huge doses they used a violent focal reaction was produced, throwing off the diseased tissue; and as only the outside of the body was affected, these pathological products were not absorbed by the blood stream, to cause further mischief, but could pass out of the system altogether. At that time, however, old tuberculin was far too risky a thing to use. Later, Finsen light was extolled, and undoubtedly constituted an advance. Still, there was a residuum of recidivistic cases which defied both surgery and Finsen light. The latter came in practice to be supplanted by *x*-rays, which were cheaper and quicker, and only very slightly less effective; although the Danish treatment is still retained in certain quarters, perhaps from sentimental reasons. Then came the newer and safer tuberculins, used in Wright's way, either under control of the opsonic index—a precaution now never adopted—or without. Wright's method still persists, perhaps. What became much commoner was the Continental method of progressive immunisation, with gradually rising dose. But one can discern the end of that too. The other day a leading dermatologist said that lupus which had been treated by tuberculin was really more obstinate than lupus not so treated. Besides, in other forms of tuberculosis the therapeutic reputation of tuberculin is gradually but surely sinking.

One sign of that occurrence is that for some years (as noted from time to time in this journal) there has been work going on in Germany to establish a chemo-therapy of tuberculosis, roughly comparable with that discovered there for syphilis. This work, in German special literature, is collectively referred to as the *Finkler'sche Heilverfahren*, from the name of its leading originator. Many substances have been, and indeed are being tried, but substances which show chemical affinity with the tubercle bacillus, conformably with Ehrlich's famous principle *Corpora non agunt nisi fixata*. Such substances have been various compounds of gold, chlorine and iodine hydrogen salts of methylene blue (for dyeing and staining are chemical processes), and compounds of copper. The word compound may be used, for there has been often added lecithin—this suggested by the work, much of it in Constantinople, of Deycke and Much. For these investigators (the originators of " partial antigens " for tubercle vaccine treatment) found that if tubercle bacilli were saturated with an emulsion of brain substance they lost their acid fastness, and were eventually dissolved altogether. The active body here was lecithin, which dissolved away the protective fatty capsule of the bacillus of Koch. Presumably then lecithin should make the bacilli more vulnerable to attack by other agents.

In connection with lupus, A. Strauss has done most. To the 1912 International Tuberculosis Congress he contributed a paper entitled " My experiences with iodine methylene blue and preparations of copper in external tuberculosis, especially lupus." Sixty cases of lupus were treated by intra-muscular injection or inunction with a copper-lecithin compound. He soon followed this with an article in the *Münchener medizinishe Wochenschrift* confirming these early good results with others. The reputation of lupus chemo-therapy seemed to grow steadily, and finally, during the war, a special institution was erected at Barmen, and Strauss appointed medical director. This event, which we, probably alone of the English medical press, noted at the time, seemed to suggest that a real advance had been made. The treatment was painful; the general verdict of German tuberculosis workers was favourable; one or two suggestions were made that the copper acted only as a local stimulant; in an abstract of the report of the German Lupus Commission which we noticed last week Strauss's institution is not specially mentioned.

All this attracted slight attention over here, and while a very few English doctors were wondering when they would be able to get some of Strauss's lekutyl, they were astonished by two papers, or perhaps more strictly speaking by one paper, in the *Lancet* of March 15. For there Dr. Gauvain of Alton, who has a high reputation for knowledge of surgical tubercle to lose, described most favourable results in lupus from the employment of a compound of basic copper sulphate with basic zinc sulphate, the invention of Dr. H. A. Ellis, tuberculosis officer for Middlesbrough. Dr. Gauvain describes this substance as being selective in its action on tuberculous tissue, as having succeeded where open-air régime and heliotherapy had not sufficed, and as being painless. Dr. Gauvain gives a very fair summary of the Continental literature of the subject, although we think his remark as to the German work owing its inspiration to an obscure French book is a patriotic *cliché*. The *Finkler'sche Heilverfahren* traces back to Ehrlich and none other. As for Dr. Ellis's paper, it amply bears out what Dr. Gauvain says, that its author was quite original in his work, did not know that such a thing as chemo-therapy of tuberculosis existed. But if Dr. Ellis has found an ointment that will cure lupus he may congratulate himself upon being a much rarer person than many a more erudite physician and better writer. All that remains to be said is that Dr. Ellis tells us where his preparations may be obtained, and something of the way he uses them.

